# Zandelisib (ME-401) in Japanese patients with relapsed or refractory indolent non-Hodgkin’s lymphoma: an open-label, multicenter, dose-escalation phase 1 study

**DOI:** 10.1007/s12185-022-03450-5

**Published:** 2022-09-15

**Authors:** Hideki Goto, Koji Izutsu, Daisuke Ennishi, Yuko Mishima, Shinichi Makita, Koji Kato, Miyoko Hanaya, Satoshi Hirano, Kazuya Narushima, Takanori Teshima, Hirokazu Nagai, Kenichi Ishizawa

**Affiliations:** 1 Department of Hematology, Hokkaido University Faculty of Medicine, N15, W7, Kita-ku, Sapporo, 060-8638 Japan; 2 Department of Hematology, National Cancer Center Hospital, Tokyo, Japan; 3 Department of Hematology and Oncology, Okayama University Hospital, Okayama, Japan; 4 Department of Hematology Oncology, The Cancer Institute Hospital, Japanese Foundation for Cancer Research, Tokyo, Japan; 5 Hematology, Oncology and Cardiovascular Medicine, Kyushu University Hospital, Fukuoka, Japan; 6 Kyowa Kirin Co., Ltd., Tokyo, Japan; 7 Department of Hematology, National Hospital Organization Nagoya Medical Center, Nagoya, Japan; 8 Division of Hematology and Cell Therapy, Department of Third Internal Medicine, Yamagata University Faculty of Medicine, Yamagata, Japan

**Keywords:** Dose escalation, Follicular lymphoma, Marginal zone lymphoma, PI3Kδ inhibitor, Zandelisib

## Abstract

The selective phosphatidylinositol 3-kinase δ inhibitor zandelisib demonstrated favorable safety and efficacy [objective response rate (ORR) 79%] in patients with B-cell malignancies in a phase 1b study in the US and Switzerland. In this phase 1 dose-escalation study (NCT03985189), 9 Japanese patients with relapsed/refractory indolent non-Hodgkin’s lymphoma (R/R iNHL) received zandelisib on a continuous daily schedule (45 or 60 mg) until progressive disease/unacceptable toxicity. No dose-limiting toxicities were observed. The maximum tolerated dose was not reached. At a median follow-up of 17.5 months, Grade ≥ 3 treatment-emergent adverse events that occurred in 2 or more patients were neutrophil count decreased (55.6%; 5/9) and diarrhea (33.3%; 3/9). Immune-related toxicities, including hepatobiliary disorder, aspartate/alanine aminotransferase increased, diarrhea/colitis, organizing pneumonia, stomatitis, and rash, led to zandelisib discontinuation in 4 patients. The investigator-assessed ORR, based on modified Lugano criteria, was 100%, including 2 complete responses (22.2%; in follicular lymphoma patients receiving 60 mg/day). Median duration of response, progression-free survival, and time to response were 7.9, 11.1, and 1.9 months, respectively. Zandelisib demonstrated a manageable safety profile at 60 mg, the recommended phase 2 dose (RP2D) in Japanese patients. The RP2D resulted in favorable pharmacokinetics and anti-tumor efficacy in Japanese patients with R/R iNHL.

*Trial registration*. NCT03985189 (ClinicalTrials.gov).

**Supplementary Information:**

The online version contains supplementary material available at 10.1007/s12185-022-03450-5.

## Introduction

Annually, B-cell non-Hodgkin’s lymphoma (NHL) is diagnosed in more than half a million patients worldwide [1]. Indolent NHL (iNHL) is a slowly progressing B-cell lymphoma that has many histological subtypes, including follicular lymphoma (FL) or marginal zone lymphoma (MZL) [2]. In recent years, various targeted therapies have been investigated to help improve outcomes in patients with relapsed or refractory (R/R) iNHL, including agents that target the phosphatidylinositol 3-kinase (PI3K) signaling pathway, which is commonly activated in hematologic and other malignancies [3].

Although 4 PI3K inhibitors, idelalisib (PI3Kδ inhibitor), copanlisib, (PI3Kα and PI3Kδ inhibitor), duvelisib (PI3Kδ and PI3Kγ inhibitor), and umbralisib (PI3Kδ and casein kinase-1ε inhibitor), have been approved in US for R/R FL [4–7], 3 compounds were voluntary withdrawn from the market. Despite therapeutic benefits, immune-related adverse events (irAEs) have limited the clinical use of PI3K inhibitors, and new agents are being investigated, particularly for long-term administration [7, 8].

The novel selective oral PI3Kδ inhibitor zandelisib (formerly PWT143 and ME-401) has a molecular structure distinct from that of other PI3Kδ inhibitors [9]. It has been granted Fast Track designation by the US Food and Drug Administration to treat patients with R/R FL who have been treated with at least 2 prior systemic therapies [10]. Zandelisib specifically inhibits the PI3Kδ signaling pathway (half maximal inhibitory concentration [IC_50_] = 0.6 nM), and its target binding (> 5 h) predicted prolonged target inhibition [11–14]. The results of a study on the inhibition of basophil activation, a marker of PI3Kδ inhibition, indicated that zandelisib 60 mg/day could achieve trough plasma concentrations associated with ≥ 90% target inhibition [15], which is expected to result in anti-tumor activity in patients. Although other PI3K inhibitors are administrated with continuous dosing, zandelisib is under development with intermittent dosing therapy (IDT; days 1–7 on, days 8–28 off) to reduce irAEs associated with PI3Kδ inhibition, without loss of efficacy.

In a phase 1b study conducted in the US and Switzerland in patients with B-cell malignancies (NCT02914938), IDT with zandelisib 60 mg was compared with continuous dosing and showed a reduction in toxicity while retaining efficacy [16]. No dose-limiting toxicities (DLTs) were reported, and zandelisib achieved a high objective response rate (ORR, 79%) and durable responses. The maximum tolerated dose (MTD) was not reached, and the recommended phase 2 dose (RP2D) was determined to be 60 mg/day [17]. However, it remains unclear whether the findings of this study can be extrapolated to Japanese patients.

To confirm the safety, tolerability, and optimal clinical dose of zandelisib in Japanese patients with R/R iNHL, this phase 1 study was conducted (NCT03985189).

## Methods

### Patients

Japanese patients aged ≥ 20 years with R/R iNHL histologically confirmed as FL, nodal MZL, extra-nodal MZL of mucosa-associated lymphoid tissue, small lymphocytic lymphoma, lymphoplasmacytic lymphoma, Waldenström’s macroglobulinemia, or equivalent, according to World Health Organization classification [2], were eligible for this study. Other key eligibility criteria included being PI3K inhibitor therapy-naïve and an Eastern Cooperative Oncology Group performance status of 0 or 1. Further details of inclusion and exclusion criteria are provided in Supplementary Table 1. Briefly, key exclusion criteria were patients who had difficulty controlling autoimmune hemolytic anemia or immune thrombocytopenia, had poorly controlled concomitant diseases, or had received systemic chemotherapy or radiotherapy within 4 weeks prior to the initiation of study treatment.

### Study design and treatment

This was a multicenter, open-label, dose-escalation, phase 1 study in Japan. All patients received oral zandelisib once daily at the same time each day, at least 1 h before or 2 h after a meal (CS: continuous schedule). Each treatment cycle was 28 days. Definitions of the DLTs described in this study can be found in Supplementary Table 2. The observation period for DLTs was from day 1 of Cycle 1 to day 1 of Cycle 2. Zandelisib was administered until disease progression or unacceptable toxicity. In the event of an AE, patients could be switched to IDT. In the case of disease progression while receiving IDT, patients could be switched back to CS per the investigator’s discretion.

A starting dose of 45 mg was set for investigating patient safety. This study adopted a standard 3 + 3 design. Zandelisib dosing was initiated in cohort 1 (*n* = 3; 45 mg/day). If DLTs occurred in 2 or more patients, the study was planned to be suspended pending a safety evaluation. If a DLT occurred in 1 patient, 3 further patients were added to cohort 1. If DLTs occurred in 0/3 or 1/6 patients in cohort 1, dosing in cohort 2 commenced (*n* = 6; 60 mg/day). If the incidence of DLTs in cohort 2 was ≤ 1/6, the maximum tolerated dose was not determined, and the dose in cohort 2 was to be selected as the recommended Japanese phase 2 dose.

Concomitant anti-cancer treatment or prophylactic medications were generally not permitted; however, patients were required to use prophylactic treatment for *Pneumocystis* pneumonia. Other permitted prophylactic treatments included those for the symptomatic treatment of AEs, such as gastritis.

This study was performed in accordance with the ethical principles of the Declaration of Helsinki, the International Conference on Harmonization Good Clinical Practice Guideline, and other applicable regulatory requirements and was approved by the Institutional Review Board at each study site. All patients provided written informed consent prior to participating in any study procedures.

### Study endpoints

The primary endpoint was the safety [number of patients with treatment-emergent adverse events (TEAEs), based on Common Terminology Criteria for Adverse Events version 5.0] and tolerability (including laboratory values, vital signs, and electrocardiogram performance) of zandelisib in Japanese patients. Hepatobiliary disorder, aspartate/alanine (AST/ALT) increased, diarrhea/colitis, organizing pneumonia, stomatitis, or rash were defined as irAEs in this manuscript. Secondary endpoints included PK (plasma concentrations and PK parameters) and the efficacy of zandelisib. Blood samples for PK evaluation were collected at pre-dose, 1.5, 3, 5, 8, 10, 12 (optional), and 24 h post-dose on Cycle 1 day 1 and Cycle 2 day 1, and at pre-dose on Cycle 1 days 8, 15, and 22. The PK parameters for Cycle 1 day 1 and Cycle 2 day 1 were calculated for each patient by non-compartmental analysis. PK parameters included maximum plasma drug concentration (*C*_max_), area under the plasma concentration–time curve (AUC) from time zero to the last measurable point (AUC_0–t_), the AUC from time zero to infinity (AUC_0-inf_), and terminal half-life (*t*_1/2_).

Efficacy endpoints included ORR; duration of response (DOR); progression-free survival (PFS); time to first response (TTR); and change in tumor size from baseline. Response was assessed by investigators based on the modified Lugano Classification lymphoma response criteria (modified Lugano criteria) [18]. If complete response (CR) was achieved by CT- or MRI-based assessment (not including bone marrow biopsy), PET/CT was performed to assess metabolic response (except for patients whose FDG-avidity was “non-avid” in the screening test). Additionally, a bone marrow biopsy was also performed if complete metabolic response was confirmed by PET/CT-based assessment.

### Statistical methods

Based on the 2 cohorts specified in the 3 + 3 design, the target number of patients was set to a maximum of 12 patients. The study was not designed for formal hypothesis testing or statistical inference. Categorical data were summarized by frequencies and percentages, and continuous data were summarized using mean and standard deviation (SD), or median and range (minimum–maximum values).

For efficacy outcomes, the Clopper–Pearson method was used to obtain 95% confidence intervals (CIs) for ORR. The Kaplan–Meier method was used to estimate the DOR and PFS for each cohort and overall. Statistical analyses were performed using SAS^®^ software version 9.4 (SAS Institute Inc., Cary, NC, USA).

## Results

### Patients

**Fig. 1:**
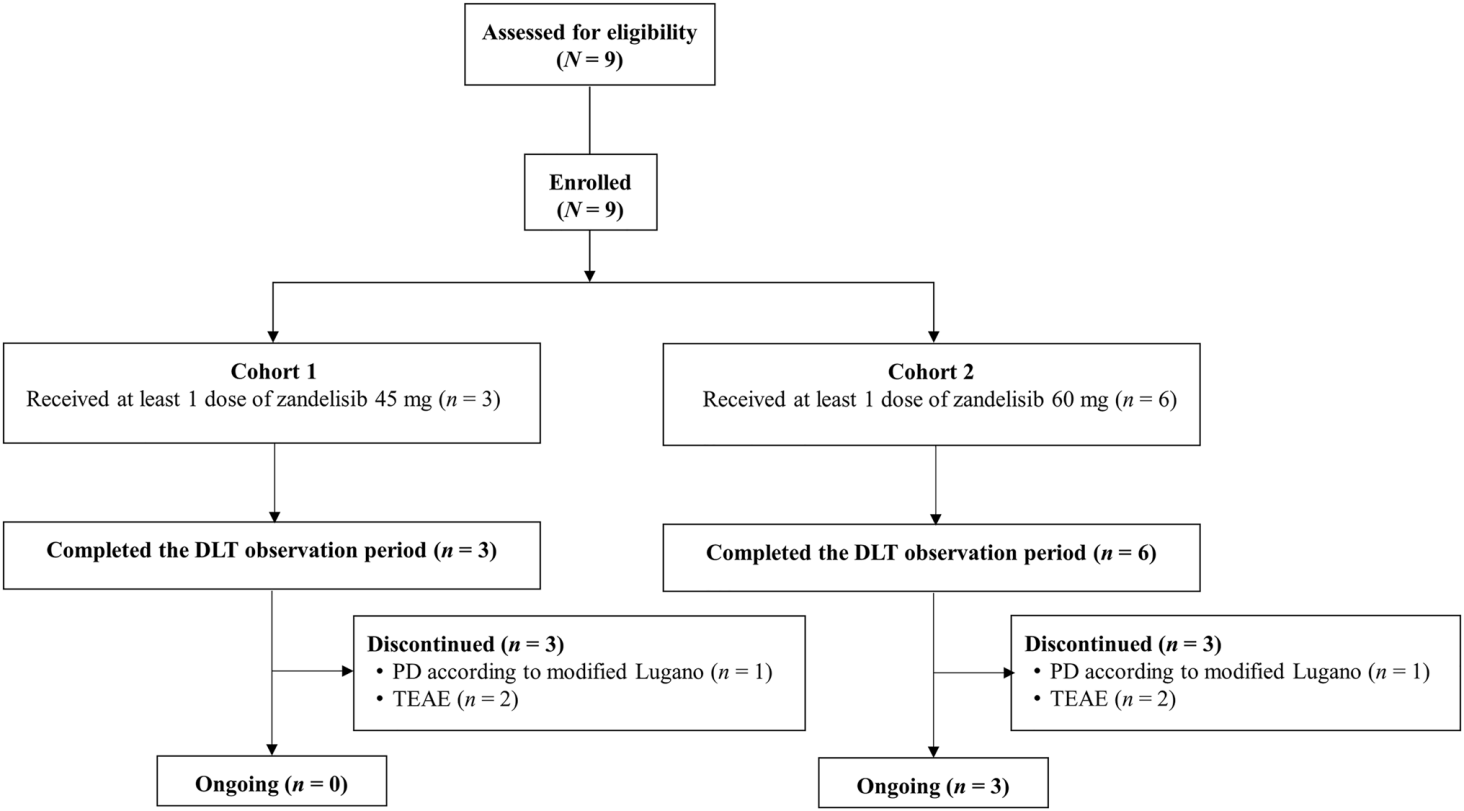
Patient disposition. *DLT* dose-limiting toxicity, *PD* progressive disease, *TEAE* treatment-emergent adverse event

From March 2019 to March 4, 2021 (data cut-off), 9 patients from 6 institutions were enrolled in the study [cohort 1 (zandelisib 45 mg/day), *n* = 3; cohort 2 (zandelisib 60 mg/day), *n* = 6; Fig. 1]. At the data cut-off date, 3 patients were continuing treatment. All patients received at least 1 dose of the study treatment and were included in the analyses. All patients in each cohort completed the DLT observation period. One patient in each cohort discontinued study treatment due to progressive disease (PD), and 2 patients in each cohort discontinued treatment due to TEAEs. Details of the incidence of TEAEs described in this study can be found in Supplementary Table 3.

At baseline, the median patient age was 69 (range 52–76) years (Table 1). Most patients (*n* = 8) had FL, and the remaining patient had nodal MZL. Overall, patients had received a median of 2 (range, 1–7) prior anti-cancer therapies. All patients had received rituximab and an alkylating agent, either alone or in combination.

**Table 1:** Baseline patient demographic and clinical characteristics

	Cohort 1 zandelisib 45 mg,* n* = 3	Cohort 2 zandelisib 60 mg, *n* = 6	Total, *N* = 9
Age, years, median (range)	69 (52–74)	67.5 (53–76)	69 (52–76)
Sex			
Female	2 (66.7)	2 (33.3)	4 (44.4)
Male	1 (33.3)	4 (66.7)	5 (55.6)
Baseline weight, kg, median (range)	52.7 (43.5–55.4)	57.8 (47.3–80.0)	55.2 (43.5–80.0)
ECOG PS			
0	2 (66.7)	6 (100)	8 (88.9)
1	1 (33.3)	0	1 (11.1)
Tumor diagnosis			
Follicular lymphoma	3 (100)	5 (83.3)	8 (88.9)
Nodal marginal zone lymphoma	0	1 (16.7)	1 (11.1)
Ann Arbor Stage classification			
I–II	1 (33.3)	1 (16.7)	2 (22.2)
III–IV	2 (66.7)	5 (83.3)	7 (77.8)
Bulky disease (> 5 cm)			
No	3 (100)	5 (83.3)	8 (88.9)
Yes	0	1 (16.7)	1 (11.1)
Number of prior cancer therapies, median (range)	1 (1–2)	2.5 (1–7)	2 (1–7)
Prior therapy			
Rituximab + alkylating agent	3 (100.0)	6 (100.0)	9 (100)
Rituximab + bendamustine	3 (100.0)	4 (66.7)	7 (77.8)
Rituximab maintenance	2 (66.7)	2 (33.3)	4 (44.4)
Rituximab + CHOP	0	5 (83.3)	5 (55.6)
Purine analog	0	1 (16.7)	1 (11.1)
Disease refractory to the most recent regimen			
No	2 (66.7)	6 (100.0)	8 (88.9)
Yes	1 (33.3)	0 (0.0)	1 (11.1)

Data are *n* (%) unless otherwise specified

*CHOP* cyclophosphamide, doxorubicin, vincristine, prednisone, *ECOG PS* Eastern Cooperative Oncology Group performance status, *FDG* fluorodeoxyglucose

**Fig. 2:**
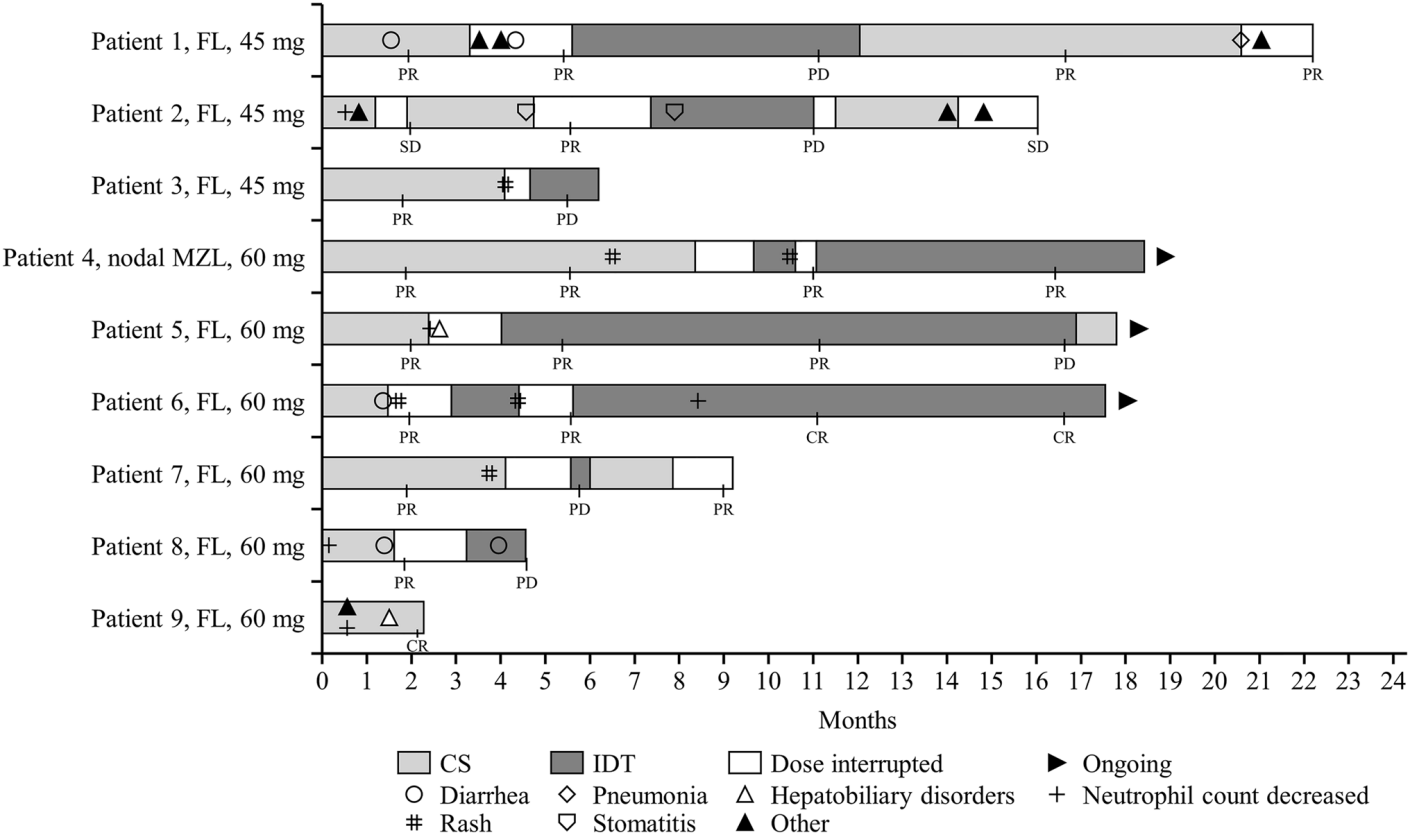
Time course of zandelisib treatment; the median duration of exposure was 14.2 months (range 1.4–20.6). Adverse events included diarrhea, pneumonia, hepatobiliary disorders, neutrophil count decreased, rash, stomatitis and other ≥ Grade 3 adverse events resulting in drug interruption, dosing schedule change, or discontinuation. *CR* complete response, *CS* continuous schedule, *FL* follicular lymphoma, *IDT* intermittent dosing treatment, *MZL* marginal zone lymphoma, *PD* progressive disease, *PR* partial response, *SD* stable disease

Figure 2 shows the time course of zandelisib treatment for individual patients.

### Safety

At the data cut-off date, the median duration of exposure was 14.2 (range, 1.4–20.6) months. No DLTs were reported, and the MTD was not reached.

The incidence of TEAEs in all patients during the study period was 100%, including 77.8% that were ≥ Grade 3; no deaths were observed. The incidences of TEAEs that occurred in at least 2 patients are shown in Table 2. Grade ≥ 3 TEAEs that occurred in more than 2 patients were neutrophil count decreased (*n* = 5, 55.6%) and diarrhea (*n* = 3, 33.3%). Granulocyte colony-stimulating factor was used in 2 patients with neutropenia (1 patient with Grade 3 and 1 patient with Grade 4 neutrophil count decreased), and zandelisib was interrupted in only 1 patient.

**Table 2:** Summary of treatment-emergent adverse events (TEAEs) occurring in ≥ 2 patients

	Cohort 1 zandelisib 45 mg, *n* = 3		Cohort 2 zandelisib 60 mg, *n* = 6		Total, *N* = 9	
System organ class	All grades	Grade ≥ 3	All grades	Grade ≥ 3	All grades	Grade ≥ 3
Preferred term	*n* (%)	*n* (%)	*n* (%)	*n* (%)	*n* (%)	*n* (%)
*TEAEs*						
Gastrointestinal disorders						
Constipation	2 (66.7)	0	2 (33.3)	0	4 (44.4)	0
Diarrhea	2 (66.7)	1 (33.3)	2 (33.3)	2 (33.3)	4 (44.4)	3 (33.3)
Nausea	1 (33.3)	0	2 (33.3)	0	3 (33.3)	0
Stomatitis	1 (33.3)	1 (33.3)	1 (16.7)	0	2 (22.2)	1 (11.1)
Vomiting	0	0	2 (33.3)	0	2 (22.2)	0
Hepatobiliary disorders						
Hepatic function abnormal	1 (33.3)	0	1 (16.7)	1 (16.7)	2 (22.2)	1 (11.1)
Immune system disorders						
Hypogammaglobulinemia	2 (66.7)	0	0	0	2 (22.2)	0
Infections and infestations						
Nasopharyngitis	2 (66.7)	0	3 (50.0)	0	5 (55.6)	0
Conjunctivitis	1 (33.3)	0	1 (16.7)	0	2 (22.2)	0
Musculoskeletal and connective tissue disorders						
Arthralgia	1 (33.3)	0	1 (16.7)	0	2 (22.2)	0
Respiratory, thoracic, and mediastinal disorders						
Oropharyngeal pain	0	0	2 (33.3)	0	2 (22.2)	0
*Investigations*						
Neutrophil count decreased	2 (66.7)	1 (33.3)	6 (100.0)	4 (66.7)	8 (88.9)	5 (55.6)
AST increased	2 (66.7)	0	3 (50.0)	0	5 (55.6)	0
WBC count decreased	2 (66.7)	0	2 (33.3)	0	4 (44.4)	0
ALT increased	2 (66.7)	0	1 (16.7)	0	3 (33.3)	0
Lymphocyte count decreased	2 (66.7)	1 (33.3)	1 (16.7)	0	3 (33.3)	1 (11.1)
CMV test positive	1 (33.3)	0	2 (33.3)	0	3 (33.3)	0
*Skin and subcutaneous tissue disorders*						
Rash maculo-papular	1 (33.3)	0	4 (66.7)	1 (16.7)	5 (55.6)	1 (11.1)
Rash	1 (33.3)	0	3 (50.0)	0	4 (44.4)	0
Dry skin	1 (33.3)	0	1 (16.7)	0	2 (22.2)	0

Data are *n* (%)

TEAEs were classified according to the Medical Dictionary for Regulatory Activities version 24.0

*ALT* alanine aminotransferase, *AST* aspartate aminotransferase, *CMV* cytomegalovirus, *WBC* white blood cell

All patients experienced Grade ≤ 3 irAEs, including hepatobiliary disorder, AST/ALT increased, diarrhea/colitis, organizing pneumonia, stomatitis, or rash. Grade 3 irAEs occurred in 7 patients (zandelisib 45 mg, *n* = 2; zandelisib 60 mg, *n* = 5), including 4 who developed these during the first 2 cycles of study treatment.

Although dosing was interrupted due to irAEs (7 patients) or neutrophil count decreased (1 patient), all these patients resumed administration with IDT (Fig. 2). Of the 7 patients who had dose interruption because of irAEs, 6 were treated with prednisolone. Recurrence of Grade ≥ 3 irAEs and any grade irAEs leading to drug interruption was observed in 4/8 patients with IDT, but no discontinuation due to irAEs occurred during IDT. Overall, 4/8 patients switched back from IDT to CS due to disease progression. Although only diarrhea (Grade 1) recurred in 1 patient, other recurrences of irAEs were not observed during CS after switchback from IDT.

Over the treatment period, 6 serious AEs (SAEs) developed in 4 patients (44.4%), all of which were Grade 3 and judged as treatment related: 1 patient with dyspnea, diarrhea, and organizing pneumonia (cohort 1), 1 patient with worsening of oral mucositis (cohort 1), 1 patient with drug-induced liver injury (cohort 2), and 1 patient with diarrhea (cohort 2). Except for the patient with dyspnea who was treated with oxygen and recovered, all patients with SAEs were treated with prednisolone. Although 3 patients discontinued due to SAEs (45 mg/day: organizing pneumonia; 45 mg/day: worsening of oral mucositis; 60 mg/day: hepatobiliary disorder), 1 of these patients resumed administration of zandelisib following completion of steroid administration. One other patient discontinued due to an irAE (60 mg/day: rash maculo-papular). No deaths were observed during treatment with zandelisib 45 mg and 60 mg.

### PK assessment

**Fig. 3:**
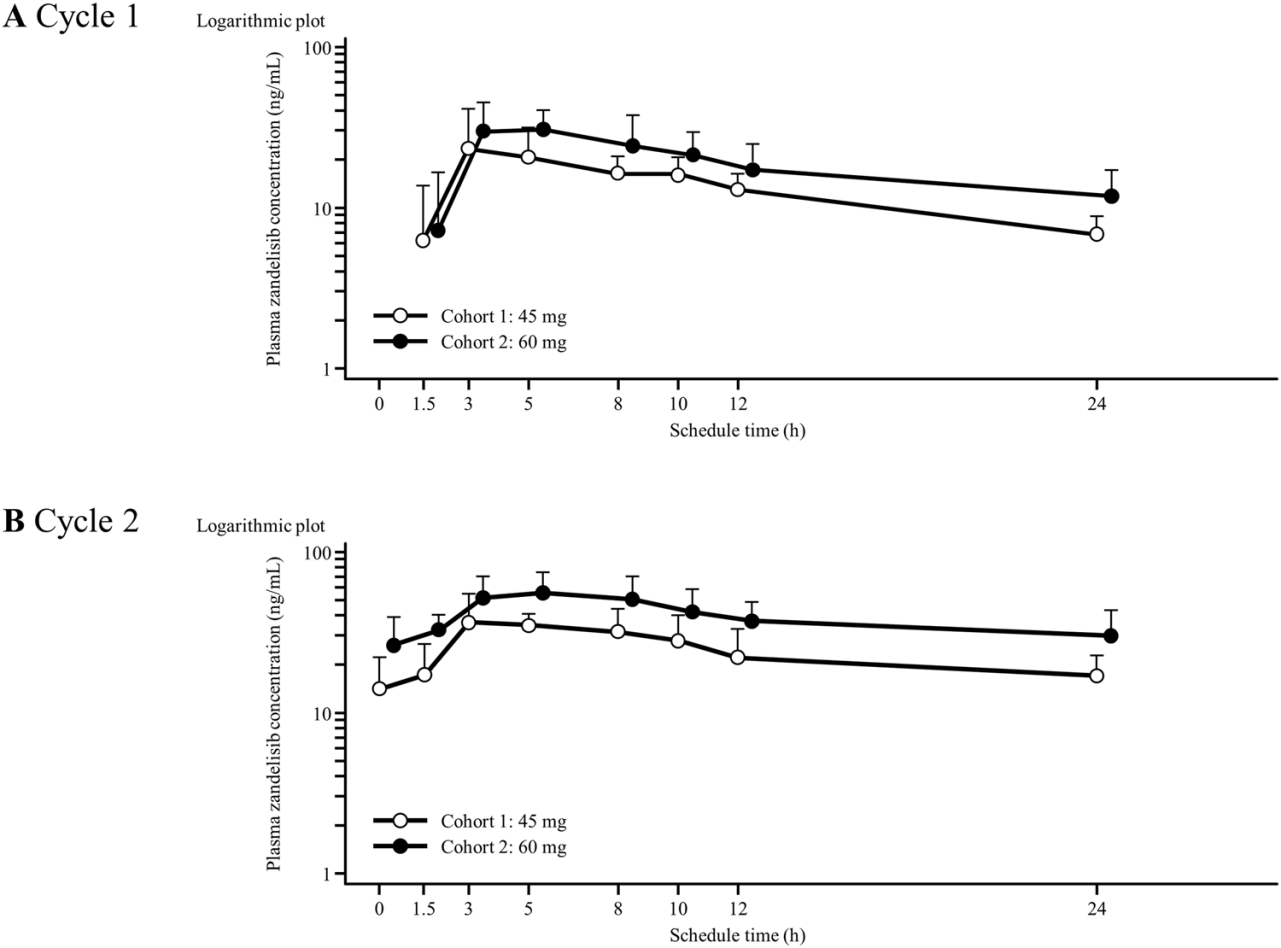
Mean (+ standard deviation) zandelisib plasma concentration–time profiles for **a** Cycle 1 (day 1 and day 2) and **b** Cycle 2 (day 1 and day 2)

The plasma concentration–time profiles of zandelisib and PK parameters are shown in Fig. 3 and Table 3. After single and multiple doses of zandelisib 45 and 60 mg, the median *t*_max_ was 3 and ~ 4.96 h, respectively. Exposure (*C*_max_ and AUC) was generally dose proportional. Exposures (*C*_max_ and AUC) on Cycle 2 Day 1 were approximately 1.5–2-fold higher than exposures on Cycle 1 Day 1. Mean ± SD pre-dose concentrations on Day 8, 15, and 22 of Cycle 1 and on Day 1 of Cycle 2 after 60 mg daily dosing were 25.2 ± 8.2, 26.1 ± 9.6, 28.2 ± 9.5, and 26.2 ± 13.1 ng/mL, respectively; PK reached steady state by Day 8. The trend was the same after 45 mg daily dosing (data not shown).

**Table 3:** Pharmacokinetic parameters of zandelisib

	Cycle	*t*_max_ (h)^a^	*C*_max_ (ng/mL)^b^	AUC_0–t_ (ng h/mL)^b^	AUC_0–inf_ (ng h/mL)^b^	*t*_1/2_ (h)^b^
Cohort 1 Zandelisib 45 mg, *n* = 3	1	3.00 (2.88–9.53)	27.5 ± 11.0	304 ± 90	479 ± 112^c^	12.5 ± 0.8^c^
	2	3.08 (2.95–7.98)	46.4 ± 6.2	601 ± 171	–	32.5 ± 10.3^c^
Cohort 2 Zandelisib 60 mg, *n* = 6	1	3.94 (3.00–7.83)	36.6 ± 13.2	435 ± 161	731 ± 331	16.4 ± 3.9
	2	4.96 (2.83–8.00)	62.8 ± 19.4	974 ± 339	–	23.5 ± 10.9^d^

*AUC* area under the plasma concentration–time curve, *AUC*_*0–t*_ AUC from time zero until time specified (24 h later), *AUC*_*0–inf*_ AUC from time zero to infinity, *C*_max_ maximum plasma concentration, *t*_max_ time of maximum plasma concentration, *t*_½_ half-life

^a^Median (min–max)

^b^Mean ± SD

^c^*n* = 2

^d^*n* = 5

### Efficacy

A summary of the anti-tumor efficacy of zandelisib is provided in Table 4; the ORR was 100%. Additionally, 8/9 patients achieved response at week 8 (after 2 administration cycles). In cohort 1, 3/3 patients achieved a best overall response of partial response (PR); in cohort 2, 2/6 patients achieved a CR, and 4/6 achieved PR. Six patients experienced PD with IDT, and 4 patients switched back to CS; 2/4 patients regained response after switching back to CS.

**Table 4:** Treatment response, as assessed by the investigator

	Cohort 1 zandelisib 45 mg, *n* = 3	Cohort 2 zandelisib 60 mg, *n* = 6	Total, *N* = 9
Objective response rate, % (95% CI)	100.0 (29.2–100.0)	100.0 (54.1–100.0)	100.0 (66.4–100.0)
Best overall response, *n* (%)			
Complete response	0 (0.0)	2 (33.3)	2 (22.2)
Partial response	3 (100.0)	4 (66.7)	7 (77.8)
Stable disease	0	0	0
Progressive disease	0	0	0

*CI* confidence interval

**Fig. 4:**
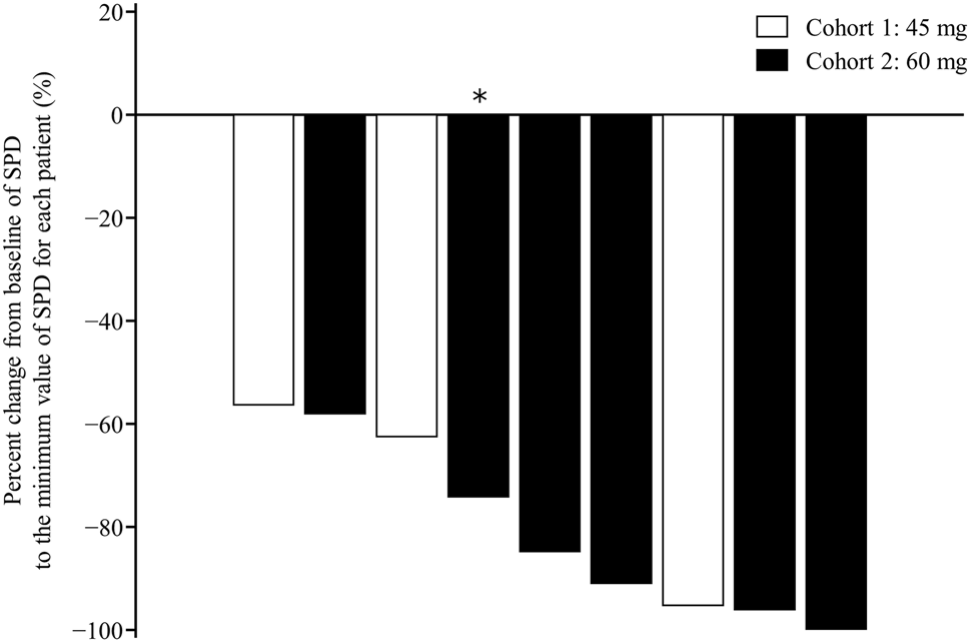
Waterfall plot of change in tumor size. *Patient with marginal zone lymphoma; all other patients are those with follicular lymphoma. *SPD* sum of perpendicular diameters

**Fig. 5:**
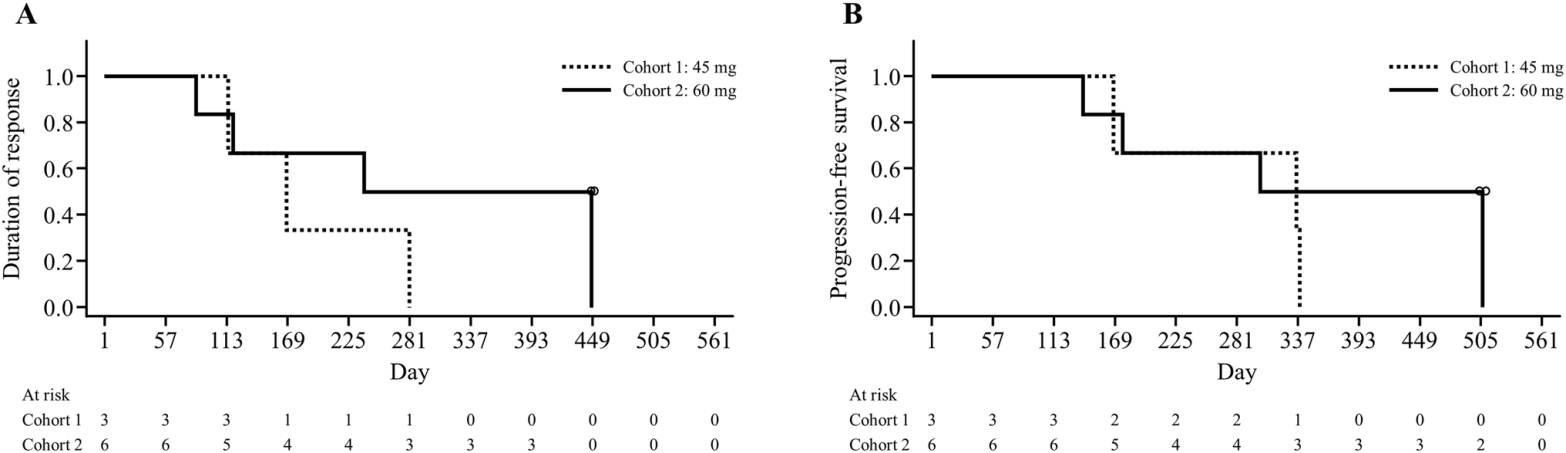
Kaplan–Meier curves of **a** duration of response and **b** progression-free survival

All patients showed a > 50% decrease from baseline in the sum of perpendicular diameters (Fig. 4). At a median follow-up of 17.5 months, the median DOR was 7.9 (95% CI 2.8–14.7) months, median PFS was 11.1 (95% CI 4.6–16.7) months, and median TTR was 1.9 (95% CI 1.8–2.1) months (Fig. 5, TTR is not shown). Median DOR (5.5 and 11.3 months) and median PFS (11.1 and 13.3 months) were longer in the 60 mg group than in the 45 mg group.

## Discussion

This is the first study evaluating the safety, tolerability and PK parameters of zandelisib in 9 Japanese patients with R/R iNHL. In this phase 1 study, zandelisib was generally well tolerated and no DLTs or fatal events were observed in both 45 and 60 mg/day doses over a median follow-up of 17.5 months. From the point of view of safety and tolerability, the RP2D in Japanese patients was found to be the same as in previous studies conducted in the US and Switzerland [17, 19]. Although dosing was interrupted due to irAEs (7 patients) or neutrophil count decreased (1 patient), all these patients resumed administration with IDT. Recurrence of Grade ≥ 3 irAEs and any grade irAEs leading to drug interruption was observed in 4/8 patients during IDT, but there was no treatment discontinuation (Fig. 2). To suppress irAEs, IDT was incorporated in the previous phase 1b study, and the incidence of Grade ≥ 3 irAEs was lower in the IDT group than in the continuous daily administration group [17]. These results suggested that IDT was tolerated at least in some patients who experienced irAEs.

These irAEs are speculated to be caused by inhibition of the pathway of normal immune cells, particularly the on-target suppression of regulatory T cells [20], and disruption of immune homeostasis. Although these irAEs were often clinically significant, patients generally recovered with treatment interruption and appropriate management, including systemic corticosteroid therapy. In fact, all SAEs defined as irAE were manageable with steroid treatment in this study. Additionally, results of previous study showed that irAEs tended to occur after 2 cycles [17, 19]. In this study, however, Grade ≥ 3 irAEs occurred in 7 patients and 4/7 patients developed them during the first 2 cycles (8 weeks) of study treatment, suggesting that patients should be monitored not only after 2 cycles but within 2 cycles in terms of the development of irAEs. Overall, the safety profile of zandelisib in Japanese patients with R/R iNHL was consistent with that observed in an ongoing three-arm phase 1b clinical trial of zandelisib conducted primarily in the US and Switzerland [17, 19].

Our results showed that exposure to zandelisib was generally dose proportional up to 60 mg/day in Japanese patients as demonstrated in healthy volunteers [15]. The results also indicate that plasma drug concentration reached steady state following 7 days of dosing. Plasma drug concentrations in patients who developed AEs during the DLT observation period were similar to those in patients who did not experience AEs (data not shown).

In terms of efficacy, the ORR of zandelisib in this study was 100% with a 22% CR rate (in 2 patients with FL receiving 60 mg/day). Overall, 8/9 patients in the whole study achieved their first response at week 8 (after 2 cycles of administration). Although the number of patients was small, this study's preliminary efficacy is consistent with the results of the phase 1b study conducted in the US and Switzerland, in which the ORR was 79% and most of anti-tumor effect was observed during the first 2 cycles (8 weeks) [17].

The dosing schedule was changed in 8 patients from CS to IDT due to toxicity, and 4 of these patients were switched back from IDT to CS when PD was evident. Of these, 2/4 patients achieved response (Fig. 2). These findings suggest that switching back from IDT to CS could result in treatment response.

Based on the emerging profile of zandelisib, both in terms of safety and efficacy on the combination schedule of CS and IDT in this study and phase 1b study (NCT02914938), zandelisib monotherapy is being assessed in patients with R/R FL and MZL in a Japanese phase 2 study [MIRAGE (NCT04533581)] and a global phase 2 study [TIDAL (NCT03768505)].

The main limitations of this phase 1 study include the small number of patients evaluated. Additionally, this study was not designed to assess the efficacy and/or optimal dosing schedule of zandelisib in Japanese patients with iNHL.

In conclusion, this Japanese phase 1 study demonstrated that zandelisib monotherapy had a favorable safety profile and positive preliminary efficacy for the treatment of Japanese patients with R/R iNHL. The RP2D was determined to be zandelisib 60 mg/day in Japanese patients. IDT was well tolerated in patients who resumed treatment after interruption due to irAEs.

## Supplementary Information

Below is the link to the electronic supplementary material.

Supplementary file1 (DOCX 48 KB)
