# Supplementary material for: Zandelisib (ME-401) in Japanese patients with relapsed or refractory indolent non-Hodgkin’s lymphoma: an open-label, multicenter, dose-escalation phase 1 study
Source: Int J Hematol. 2022 Sep 15;116(6):911–21. doi: 10.1007/s12185-022-03450-5 (PMC9668928; doi:10.1007/s12185-022-03450-5)
Supplement: Supplementary file 1 — Supplementary file1 (DOCX 48 KB) [file 12185_2022_3450_MOESM1_ESM.docx]

**Supplementary Materials**

**Supplementary Table 1.** Inclusion and exclusion criteria for study enrollment

| **Inclusion criteria** |
| --- |
| - Provided written informed consent |
| - Aged ≥ 20 years at the time of providing written informed consent |
| - Relapsed or refractory indolent B-cell non-Hodgkin lymphoma histologically diagnosed as follicular lymphoma, nodal marginal zone lymphoma, mucosa-associated lymphoid tissue lymphoma, small lymphocytic lymphoma, lymphoplasmacytic lymphoma, Waldenstrom macroglobulinemia, or similar according to WHO classification |
| - PI3K therapy naïve |
| - Received Bruton’s tyrosine kinase inhibitors with no disease progression during use |
| - Previously received ≥ 1 systemic chemotherapy (including anti-CD20 antibodies and chemotherapy) and judged by the principal investigator or the sub-investigator to require new treatment, including patients for whom standard systemic chemotherapy treatment options are available |
| - Nodular lesions with longest diameter of ≥ 1.5 cm on computed tomography or magnetic resonance imaging; lesions previously treated with radiotherapy, that were subject to biopsy sampling, or bone lesions are not included |
| - Eastern Cooperative Oncology Group Performance Status 0 or 1 |
| - Hematopoietic capacity and hepatic, renal, and cardiac function meeting all of the following criteria in the preliminary examination; additionally, supportive care (e.g., administration of granulocyte colony-stimulating factor products or blood transfusions) that may affect the following examinations shall not be implemented within 14 days prior to the pretest:   ANC: ≥ 1000/μL  PLT: ≥ 7.5 × 104/μL  Hb-level: ≥ 8.0 g/dL  AST and ALT: ≤ 2.5 times the upper limit of the reference level of the trial site  T-Bil: ≤ 1.5 times the upper limit of the reference level of the trial site; in the case of patients diagnosed with Gilbert’s syndrome, the value is ≤ 3 times the upper limit of the reference level of the institution  Cr: ≤ 1.5 times the upper limit of the reference level of the trial site or eGFR not less than 50 mL/min; eGFR will be calculated from the following equations for eGFR in Japanese patients: Age is the age at each assessment point.  eGFR = 194 × serum creatinine ^−1.094^× age ^−0.287^ (for women, multiply left equation by 0.739) |
| - Individuals with pre-test corrected QT-interval (QTcF) ≤ 450 msec; if QTc prolongation is caused by right bundle branch block and the waveform is stable, the QTc is < 480 msec |
| - An echocardiographic left ventricular ejection fraction > 50% at the pretest |
| - For women of childbearing potential, those with a negative pretest pregnancy test |
| - Consent to participate in the study and consent to contraception for at least 90 days after completion of administration of the investigational product |
| - Agreed not to donate sperm or egg cells within 90 days after the completion of administration of the investigational drug from the consent to participate in the clinical trial |
| **Exclusion criteria** |
| - Major surgical procedure within 4 weeks of the start of investigational drug administration |
| - Autoimmune hemolytic anemia or immune thrombocytopenia that is difficult to control |
| - Difficult-to-control disease, e.g., active infection, hypertension, angina pectoris, arrhythmia, lung disease, digestive system disease, skin disease, autoimmune disease or immune dysfunction |
| - Test positive for either HBV antigen or antibody, HCV antibody, HIV antibody, or HTLV-1 antibody; however, participation in this clinical trial is permitted in the following cases:   HBV test at pretest showing negative HBs Ag and positive for either HBc antibody or HBs antibody or both, but below the lower limit of quantitation or < 20 IU/mL (1.3 LogIU/mL) by HBV-DNA test (including those below the lower limit of quantitation or < 20 IU/mL (1.3 LogIU/mL) by administration of nucleic acid analogs (e.g., entecavir or tenofovir)  HBs antibody alone positive by hepatitis B vaccination  Patients with positive HCV antibodies but negative HCV nucleic acid amplification test (HCV-RNA test) in the HCV antibody test at the pretest |
| - Active or a history of interstitial lung disease (including drug-induced pneumonitis and radiation pneumonitis) |
| - Malignancies, other than those specified in the inclusion criteria, within 2 years before informed consent, or with overlapping cancers of other activity than those in the inclusion criteria; however, patients with hormonal therapy for prostate cancer or noninvasive cancers such as cervical carcinoma in situ, nonmelanocytic skin cancer, ductal carcinoma in situ, and gastric intramucosal cancer who have been cured by surgery, etc., may participate in this clinical trial |
| - History of clinically significant cardiovascular anomalies such as congestive heart failure (New York Heart Association functional class II or higher) and myocardial infarction within 6 months prior to obtaining informed consent |
| - Gastrointestinal diseases judged to affect the absorption of the investigational product (e.g., Crohn’s disease, resection of small or large intestine, ulcerative colitis, or malabsorption syndrome) |
| - Adverse events (excluding alopecia and laboratory abnormalities within the range described in the inclusion criteria) that have not recovered below Grade 1 due to previously administered anticancer drugs; however, in the event of a patient with a stable symptom and an irreversible adverse event that is obviously not aggravated by the investigational product (e.g., loss of hearing), participation in the study may be permitted at the discretion of the principal investigator or the subinvestigator |
| - History of transplantation therapy, such as hematopoietic stem cell transplantation; however, patients who undergo autologous peripheral blood stem cell transplantation before 3 months of obtaining informed consent may participate in this clinical trial |
| - Received other investigational products, systemic chemotherapy, or radiotherapy within 4 weeks before the start of administration of the investigational product |
| - Breastfeeding women |
| - Mental illness or social situations that may affect compliance with the study or obtaining informed consent |
| - Judged unfavorable to participate in the study by the principal investigator or the subinvestigator |

*Ag* antigen, *ALT* alanine aminotransferase, *ANC* neutrophil count, *AST* aspartate aminotransferase, *Cr* creatinine, *eGFR* estimated glomerular filtration rate, *Hb* hemoglobin, *HBV* hepatitis B virus, *HCV* hepatitis C virus, *HIV* human immunodeficiency virus, *HTLV-1* human T-cell leukemia virus type 1, *PI3K* phosphatidylinositol 3-kinase inhibitor, *PLT* platelet count, *QT interval* time from the onset of the Q wave to the end of the T wave in an electrocardiogram, *QTc interval* QT-interval corrected for heart rate, *QTcF* QT-interval corrected by QTcB, Fridericia method corrected by Bazett method, *T-Bil* total bilirubin

**Supplementary Table 2.** Definition of dose-limiting toxicities (DLTs)

| - DLTs that occurred during the 28-day DLT observation period (from the start of administration of the investigational drug, Day 1 of Cycle 1, to Day 1 of Cycle 2) were tabulated using the Medical Dictionary for Regulatory Activities version 24.0, and the severity was determined using the Common Terminology Criteria for Adverse Events version 5.0 - A DLT was defined as a clinically significant, as determined by the investigator, hematologic or non-hematologic AE that occurred during the DLT observation period, an AE that led to discontinuation despite appropriate treatment, or an AE that required withdrawal of zandelisib on 8 or more occasions - Hematologic events included:   - Grade 4 thrombocytopenia or thrombocytopenia requiring platelet transfusion   - Grade 4 anemia or anemia requiring red blood cell transfusion   - Grade 4 neutrophil count reduction   - Grade 4 febrile neutropenia - Non-hematologic events included:   - Grade 3 AEs that persisted despite outpatient standard symptomatic treatment |
| --- |

*AE* adverse event

**Supplementary Table 3.** Incidence of treatment-emergent adverse events (TEAEs) in the whole study by System Organ Class and Preferred Term

| **System Organ Class**  **Preferred Term** | **Cohort 1 zandelisib 45 mg, *n* = 3** | |  | **Cohort 2 zandelisib 60 mg, *n* = 6** | |  | **Total, *N* = 9** | |
| --- | --- | --- | --- | --- | --- | --- | --- | --- |
|  | **All grades** | **Grade ≥ 3** |  | **All grades** | **Grade ≥ 3** |  | **All grades** | **Grade ≥ 3** |
|  | ***n* (%)** | ***n* (%)** |  | ***n* (%)** | ***n* (%)** |  | ***n* (%)** | ***n* (%)** |
| **TEAEs** |  |  |  |  |  |  |  |  |
| Blood and lymphatic system disorders | 1 (33.3) | 0 |  | 0 | 0 |  | 1 (11.1) | 0 |
| Anemia | 1 (33.3) | 0 |  | 0 | 0 |  | 1 (11.1) | 0 |
| Cardiac disorders | 1 (33.3) | 0 |  | 0 | 0 |  | 1 (11.1) | 0 |
| Bradycardia | 1 (33.3) | 0 |  | 0 | 0 |  | 1 (11.1) | 0 |
| Ear and labyrinth disorders | 1 (33.3) | 0 |  | 0 | 0 |  | 1 (11.1) | 0 |
| Tinnitus | 1 (33.3) | 0 |  | 0 | 0 |  | 1 (11.1) | 0 |
| Eye disorders | 1 (33.3) | 0 |  | 1 (16.7) | 0 |  | 2 (22.2) | 0 |
| Blepharitis | 0 | 0 |  | 1 (16.7) | 0 |  | 1 (11.1) | 0 |
| Dry eye | 1 (33.3) | 0 |  | 0 | 0 |  | 1 (11.1) | 0 |
| Gastrointestinal disorders | 2 (66.7) | 2 (66.7) |  | 4 (66.7) | 2 (33.3) |  | 6 (66.7) | 4 (44.4) |
| Constipation | 2 (66.7) | 0 |  | 2 (33.3) | 0 |  | 4 (44.4) | 0 |
| Diarrhea | 2 (66.7) | 1 (33.3) |  | 2 (33.3) | 2 (33.3) |  | 4 (44.4) | 3 (33.3) |
| Nausea | 1 (33.3) | 0 |  | 2 (33.3) | 0 |  | 3 (33.3) | 0 |
| Stomatitis | 1 (33.3) | 1 (33.3) |  | 1 (16.7) | 0 |  | 2 (22.2) | 1 (11.1) |
| Vomiting | 0 | 0 |  | 2 (33.3) | 0 |  | 2 (22.2) | 0 |
| Abdominal discomfort | 0 | 0 |  | 1 (16.7) | 0 |  | 1 (11.1) | 0 |
| Abdominal pain upper | 1 (33.3) | 0 |  | 0 | 0 |  | 1 (11.1) | 0 |
| Pancreatitis | 1 (33.3) | 1 (33.3) |  | 0 | 0 |  | 1 (11.1) | 1 (11.1) |
| General disorders and administration site conditions | 1 (33.3) | 0 |  | 0 | 0 |  | 1 (11.1) | 0 |
| Infusion site extravasation | 1 (33.3) | 0 |  | 0 | 0 |  | 1 (11.1) | 0 |
| Hepatobiliary disorders | 1 (33.3) | 0 |  | 2 (33.3) | 2 (33.3) |  | 3 (33.3) | 2 (22.2) |
| Hepatic function abnormal | 1 (33.3) | 0 |  | 1 (16.7) | 1 (16.7) |  | 2 (22.2) | 1 (11.1) |
| Drug-induced liver injury | 0 | 0 |  | 1 (16.7) | 1 (16.7) |  | 1 (11.1) | 1 (11.1) |
| Immune system disorders | 2 (66.7) | 0 |  | 1 (16.7) | 0 |  | 3 (33.3) | 0 |
| Hypogammaglobulinemia | 2 (66.7) | 0 |  | 0 | 0 |  | 2 (22.2) | 0 |
| Drug hypersensitivity | 0 | 0 |  | 1 (16.7) | 0 |  | 1 (11.1) | 0 |
| Infections and infestations | 2 (66.7) | 0 |  | 4 (66.7) | 0 |  | 6 (66.7) | 0 |
| Nasopharyngitis | 2 (66.7) | 0 |  | 3 (50.0) | 0 |  | 5 (55.6) | 0 |
| Conjunctivitis | 1 (33.3) | 0 |  | 1 (16.7) | 0 |  | 2 (22.2) | 0 |
| Cytomegalovirus infection | 0 | 0 |  | 1 (16.7) | 0 |  | 1 (11.1) | 0 |
| Herpes simplex | 1 (33.3) | 0 |  | 0 | 0 |  | 1 (11.1) | 0 |
| Herpes zoster | 0 | 0 |  | 1 (16.7) | 0 |  | 1 (11.1) | 0 |
| Subcutaneous abscess | 0 | 0 |  | 1 (16.7) | 0 |  | 1 (11.1) | 0 |
| Cytomegalovirus viremia | 1 (33.3) | 0 |  | 0 | 0 |  | 1 (11.1) | 0 |
| Oral herpes | 1 (33.3) | 0 |  | 0 | 0 |  | 1 (11.1) | 0 |
| Metabolism and nutrition disorders | 1 (33.3) | 1 (33.3) |  | 0 | 0 |  | 1 (11.1) | 1 (11.1) |
| Decreased appetite | 1 (33.3) | 1 (33.3) |  | 0 | 0 |  | 1 (11.1) | 1 (11.1) |
| Musculoskeletal and connective tissue disorders | 2 (66.7) | 0 |  | 2 (33.3) | 0 |  | 4 (44.4) | 0 |
| Arthralgia | 1 (33.3) | 0 |  | 1 (16.7) | 0 |  | 2 (22.2) | 0 |
| Back pain | 1 (33.3) | 0 |  | 0 | 0 |  | 1 (11.1) | 0 |
| Flank pain | 0 | 0 |  | 1 (16.7) | 0 |  | 1 (11.1) | 0 |
| Joint effusion | 1 (33.3) | 0 |  | 0 | 0 |  | 1 (11.1) | 0 |
| Muscle spasms | 1 (33.3) | 0 |  | 0 | 0 |  | 1 (11.1) | 0 |
| Nervous system disorders | 1 (33.3) | 0 |  | 0 | 0 |  | 1 (11.1) | 0 |
| Dysgeusia | 1 (33.3) | 0 |  | 0 | 0 |  | 1 (11.1) | 0 |
| Hypoesthesia | 1 (33.3) | 0 |  | 0 | 0 |  | 1 (11.1) | 0 |
| Psychiatric disorders | 1 (33.3) | 0 |  | 0 | 0 |  | 1 (11.1) | 0 |
| Insomnia | 1 (33.3) | 0 |  | 0 | 0 |  | 1 (11.1) | 0 |
| Renal and urinary disorders | 1 (33.3) | 0 |  | 0 | 0 |  | 1 (11.1) | 0 |
| Renal impairment | 1 (33.3) | 0 |  | 0 | 0 |  | 1 (11.1) | 0 |
| Respiratory, thoracic, and mediastinal disorders | 1 (33.3) | 1 (33.3) |  | 3 (50.0) | 0 |  | 4 (44.4) | 1 (11.1) |
| Oropharyngeal pain | 0 | 0 |  | 2 (33.3) | 0 |  | 2 (22.2) | 0 |
| Cough | 0 | 0 |  | 1 (16.7) | 0 |  | 1 (11.1) | 0 |
| Dyspnea | 1 (33.3) | 1 (33.3) |  | 0 | 0 |  | 1 (11.1) | 1 (11.1) |
| Organizing pneumonia | 1 (33.3) | 1 (33.3) |  | 0 | 0 |  | 1 (11.1) | 1 (11.1) |
| Pharyngeal paresthesia | 0 | 0 |  | 1 (16.7) | 0 |  | 1 (11.1) | 0 |
| Skin and subcutaneous tissue disorders | 3 (100.0) | 0 |  | 6 (100.0) | 1 (16.7) |  | 9 (100.0) | 1 (11.1) |
| Rash maculopapular | 1 (33.3) | 0 |  | 4 (66.7) | 1 (16.7) |  | 5 (55.6) | 1 (11.1) |
| Rash | 1 (33.3) | 0 |  | 3 (50.0) | 0 |  | 4 (44.4) | 0 |
| Dry skin | 1 (33.3) | 0 |  | 1 (16.7) | 0 |  | 2 (22.2) | 0 |
| Eczema | 0 | 0 |  | 1 (16.7) | 0 |  | 1 (11.1) | 0 |
| Erythema multiforme | 0 | 0 |  | 1 (16.7) | 0 |  | 1 (11.1) | 0 |
| Pruritus | 0 | 0 |  | 1 (16.7) | 0 |  | 1 (11.1) | 0 |
| Urticaria | 1 (33.3) | 0 |  | 0 | 0 |  | 1 (11.1) | 0 |
| **Investigations** | 3 (100.0) | 2 (66.7) |  | 6 (100.0) | 4 (66.7) |  | 9 (100.0) | 6 (66.7) |
| Neutrophil count decreased | 2 (66.7) | 1 (33.3) |  | 6 (100.0) | 4 (66.7) |  | 8 (88.9) | 5 (55.6) |
| AST increased | 2 (66.7) | 0 |  | 3 (50.0) | 0 |  | 5 (55.6) | 0 |
| WBC count decreased | 2 (66.7) | 0 |  | 2 (33.3) | 0 |  | 4 (44.4) | 0 |
| ALT increased | 2 (66.7) | 0 |  | 1 (16.7) | 0 |  | 3 (33.3) | 0 |
| Lymphocyte count decreased | 2 (66.7) | 1 (33.3) |  | 1 (16.7) | 0 |  | 3 (33.3) | 1 (11.1) |
| CMV test positive | 1 (33.3) | 0 |  | 2 (33.3) | 0 |  | 3 (33.3) | 0 |
| Amylase increased | 1 (33.3) | 0 |  | 0 | 0 |  | 1 (11.1) | 0 |
| Blood creatinine increased | 0 | 0 |  | 1 (16.7) | 0 |  | 1 (11.1) | 0 |
| Eosinophil count increased | 1 (33.3) | 0 |  | 0 | 0 |  | 1 (11.1) | 0 |
| GGT increased | 0 | 0 |  | 1 (16.7) | 1 (16.7) |  | 1 (11.1) | 1 (11.1) |

Data are *n* (%)

TEAEs were classified according to the Medical Dictionary for Regulatory Activities version 24.0

*ALT* alanine aminotransferase, *AST* aspartate aminotransferase, *CMV* cytomegalovirus, *WBC* white blood cell,

*GGT* Gamma-glutamyl transferase
